# Dysglycemia in Critically Ill Children Admitted to Jimma Medical Centre, Southwest Ethiopia

**DOI:** 10.4314/ejhs.v31i2.14

**Published:** 2021-03

**Authors:** Habtamu Sime, Melkamu Berhane, Tsion Tilahun, Temam Kedir, Diriba Dereje, Muktar Beshir, Iyasu Tadesse

**Affiliations:** 1 Department of Pediatrics and Child Health, Jimma University; 2 Department of Medical Physiology, Jimma University; 3 Department of Epidemiology, Jimma University; 4 Australian Centre for Precision Health, University of South Australia Cancer Research Institute, SA, Australia; 5 School of Clinical and Health Sciences, University of South Australia, SA, Australia

**Keywords:** Dysglycemia, critically ill children, hypoglycemia, Hyperglycemia

## Abstract

**Background:**

Abnormal blood glucose level is one of the most frequently encountered problems in children with severe illnesses. However, its magnitude and outcome have rarely been determined in Ethiopia. We aimed to determine the magnitude, associated factors and outcome of dysglycemia in critically ill children admitted to Jimma Medical Center.

**Methods:**

Prospective longitudinal study was conducted on children aged 28 days to 14 years admitted with critical illnesses at the different units of the Department of Pediatrics and Child Health of Jimma Medical Center, Southwest Ethiopia, from June to August 2019. Data were collected by trained medical personnel using structured questionnaire and analyzed using Statistical Package for Social Sciences (SPSS) windows version 20.0. Dysglycemia was considered whenever the child had a random blood sugar >150mg/dl or <45mg/dl.

**Result:**

Dysglycemia was seen at admission in 139/481, 28.9% children; 24 (5.0%) had hypoglycemia whereas 115 (23.9%) had hyperglycemia. The factors associated with dysglycemia at admission were severe acute malnutrition (p=002, AOR=3.09, CI=1.18,7.77), impaired mental status (p=0.003, AOR=4.63, CI=1.68, 12.71), place of residence (p=0.01, AOR=1.85, CI=1.15–2.96) and presence of diarrhea on date of admission. Among the children who had dysglycemia at admission, 16/139, 11.5% died.

**Conclusion:**

Dysglycemia is a common problem in critically ill children in the setting. Blood glucose level should be determined for all critically ill children, and routine empirical administration of dextrose should be minimized since most of the children with dysglycemia had hyperglycemia than hypoglycemia.

## Introduction

Deaths in hospital often occur within 24 hours of admission. Many of these deaths could be prevented if very sick children are identified soon after their arrival to the health facility and treatment is started immediately. Therefore, a process of rapid triage for all children presenting to health facilities needs to be put in place to determine whether any emergency or priority signs are present. According to the World Health Organization's (WHO) emergency triage assessment and treatment (ETAT) and integrated management of neonatal and childhood illnesses (IMNCI) guidelines, a child is said to have critical illness if he/she has any of the following: inability to drink or drinking poorly, vomiting of everything, convulsions, lethargy or unconsciousness, severe dehydration, severe acute malnutrition, obstructed or absent breathing, respiratory distress, signs of shock (cold extremities with capillary refill time >3s and weak and fast pulse) and active bleeding requiring transfusion (1,2).

The term dysglycemia describes the form of hyperglycemia, hypoglycemia, and/or marked glucose variability. In recent years, glycemic variability has gained importance among critically ill patients; however, there is no consensus reached on the definition of glucose variability. However, it has traditionally been described as any patient who had both a hyperglycemic and a hypoglycemic measurement during the first 7 days of intensive care unit (ICU) stay or during a single ICU admission (3). According to WHO, outside the neonatal period, hypoglycemia is defined as blood glucose <45 mg/dl (<2.5 mmol/L) in a well nourished child or <55 mg/dl (3 mmol/L) in a malnourished child (4), whereas hyperglycemia is defined as blood glucose greater than 125 mg/dL while fasting and greater than 180 mg/dL 2 hours postprandial (4).

Alteration of blood glucose homeostasis (dysglycemia) is common in critically ill children. Particularly, dysglycaemia is commonly encountered in patients with severe malaria, acute respiratory distress, septicemia, diarrhea and vomiting (5,6). Additionally, the risk of hyperglycemia is highest amongst patients recovering from cardiac surgery, traumatic brain injury, major burns and sepsis. Moreover, mechanical ventilation, extracorporeal support and vaso active drug support further enhance the risk of developing hyperglycemia (3). However, the episodes of severe hypoglycemia occurring spontaneously during the management of critically ill patients are rare (usually observed in less than 1.5% of patients); patients with liver disease (fulminant hepatic failure), septic shock with adrenal failure, malaria and malnutrition are at high risk (3,9).

During stress to the body in the form of an acute illness, there is an increased cortisol and catecholamine secretion due to activation of the hypothalamic-pituitary adrenal axis and sympathetic system. These stress hormones, like epinephrine and cortisol, and the inflammatory cytokines stimulate gluconeogenesis and glycogenolysis while hampering glucose uptake by peripheral tissues, leading to hyperglycemia (3). Hypoglycemia in critically ill children can be either a result of the failure of fasting adaptation of the body or iatrogenic (3,9).

In studies done in Laos and Madagascar, the overall prevalence of dysglycaemia was found to be 42.5 % and 34.1% respectively (4,9). It is estimated that stress hyperglycemia with blood glucose concentration >150 mg/dl occurs in 49–72% of critically ill children, whereas blood glucose concentrations higher than 200 mg/dl are recorded in as many as 20–35% of them (7). However, in a study done in our hospital, 6% of children admitted to the pediatrics ward had an abnormal blood glucose level on admission, 4.8% and 1.2% of them having hyperglycemia and hypoglycemia respectively (13).

Hyperglycemia has deleterious effects as it results in neutrophil and cytokine dysfunction, impairing nitric oxide generation which ultimately leads to immune dysfunction. Additionally, it augments coagulation by increasing the expression of factor III (tissue factor) and factor VIIA (activated factor VII). This increased coagulation activity leading to micro thrombosis results in multi-organ failure (3).

Various studies have shown the association of glucose variability with increased mortality. A study done in India showed that patients with isolated blood glucose variability were 5.4 times more likely to die than those with no blood glucose abnormality (8,11). Besides increased mortality, dysglycemic patients are more likely to develop complications which include worsening organ function, multiorgan dysfunction syndrome and long term consequences like neurologic damage resulting in mental retardation, developmental delay, recurrent seizures and personality disorders associated with hypoglycemia (5,10–12). Additionally, both hyperglycemia and glucose variability are associated with long pediatric ICU (PICU) stay (11).

Despite these facts, the prevalence, associated factors and outcome of dysglycemia is not well studied in Ethiopia in general and Jimma in particular. Hence, this study was done to fill this gap with an overall aim of determining the magnitude, associated factors and outcome of dysglycaemia in critically ill children admitted to Jimma Medical Center, Southwest Ethiopia.

## Methods and Materials

**Study area and period**: The study was done in Jimma Medical Center, the only teaching and referral hospital in the southwestern part of the country from June to August 2019. The study participants were enrolled from the different units under the Department of Pediatrics and Child Health including PICU, pediatric emergency unit, critical care rooms (high dependency unit) and nutritional rehabilitation unit (NRU).

**Study design and population**: A prospective longitudinal study was done on all critically ill children aged 28 days to 14 years admitted to the different units of the Department of Pediatrics and Child Health during the study period. Critical illness was defined as the presence of any severe problem with the airway, breathing or circulation, or acute deterioration of conscious state, including apnoea, upper airway obstruction, hypoxaemia, central cyanosis, severe respiratory distress, total inability to feed, shock, severe dehydration, active bleeding requiring transfusion, unconsciousness or seizures (1). Children who had been on steroid for more than 48 hours, children who were on beta agonist, children receiving intravenous (IV) glucose therapy before their arrival, children with history of diabetes mellitus and children who were suspected to have inborn errors of metabolism were excluded from the study.

**Data collection procedure**: Relevant data including socio-demographic characteristics, clinical history, length of stay in the hospital and clinical outcome were collected from all children using a structured case recording format. Severity of illness was measured by using WHO pediatric ETAT plus guideline (15). All children admitted to the different units with acute illnesses had their blood glucose determined at admission, every four hours for the first 24 hours and then every 8 hours for the next 48 hours. We used a drop of whole blood (finger prick) and a point-of-care bed side glucometer (*i*-Quare DS-W Alliance International Co., New Taipei, Taiwan) which measures a range of glucose concentrations between 1.2–33.3mmol/L (20–600mg/dL). We defined hyperglycemia and hypoglycaemia as blood glucose greater than 8.3mmol/L (150mg/dL) and less than 2.5mmol/L(45mg/dL) respectively, whereas gluc ose variability was defined as the occurrence of both hypoglycemia and hyperglycemias in the first 7 days of hospital stay. All the children enrolled into the study were followed up to the time of death or discharge from the hospital. Children with dysglycemia were managed by the treating team according to the protocol at the hospital.

**Statistical analysis**: Data were entered into EpiData Manager 4.0.2 (Odense, Denmark) and exported to SPSS Version 20.0 (SPSS, Chicago, IL, USA) for statistical analysis. Based on serial blood glucose level, patients were divided into four groups: “only hyperglycemia group” (having at least one hyperglycemia episode), “only hypoglycemia group” (having at least one hypoglycemia episode), “glucose variability” (having both hypoglycemia and hyperglycemia episodes), and “normoglycemia” (all glucose measurements in normal range). All groups were compared with each other with demographic variables like age, sex, weight, admission symptoms, nutritional status, final diagnosis, and vital signs at admission. Binary logistic regression analysis was done to see the associations between the dependent and independent variables. Variables with p-value <0.25 on bivariate logistic regression were further analyzed by multivariate logistic regression. The results of regression model were presented as adjusted odds ratio with 95% confidence intervals.

**Ethical considerations**: Ethical approval was obtained from Jimma University Institutional Review Board. Written informed consent from parents/guardians and assent from older children were obtained before enrollment to the study. The results of the random blood glucose were provided to the treating physicians immediately, and children with dysglycemia were treated by the treating physicians as per the hospital's guideline.

## Results

During the study period, a total of 1955 children were seen at the different units. Out of this, 1474 were excluded because they did not fulfill the inclusion criteria, and a total of 481 children were enrolled into the study.

**Socio demographic characteristics:** The majority (203, 42.2%) of the children were less than 12 months, and over half (269, 55.9%) of the children were males. Closer to two third (306, 63.6%) of the respondents were coming out side of Jimma Town, and half of the mothers or care givers (243, 50.5%) of the children were house wives (Table 1).

**Table 1:** Socio demographic characteristics of mothers/caregivers and critically ill children admitted to JMC, Southwest Ethiopia, 2019 (N=481)

Variables	Category	Frequency (N, %)
**Age(months)**	< 12 months	203(42.2)
	12–59 months	180(37.4)
	≥60 months	98(20.4)
**Sex**	Female	212(44.1)
	Male	269(55.9)
**Residence of parents/care givers**	Jimma Town	175(36.4)
	Out of Jimma Town	306(63.6)
Occupational status of the	House wife	243(50.5)
mothers	Farmers	102(21.2)
	Self employed	75(15.6)
	Government employee or company	61(12.7)
**Educational status of**	Illiterate	**169(35.1)**
**mother/care giver**	Read and write	75(15.6)
	Primary	77(16.0)
	Secondary	73(15.2)
	High school and above	87(18.1)
Monthly income of the care	Less 2000 ETB	246(51.1)
takers/mothers	More than 2000 ETB	235(48.9)

**Medical characteristics of the children**: The majority (366, 76.1%) of the children in this study had respiratory distress followed by inability to drink or drinking poorly (215, 44.7%). Only very few patients (13, 2.7%) had shock (Table 2).

**Table 2:** Medical characteristics of children at admission to JMC, Southwest Ethiopia, 2019 (N=481)

Medical characteristics	Category	Frequency (N, %)
Respiratory distress	Yes	366(76.1)
	No	115(23.9)
Inability to drink	Yes	215(44.7)
	No	266(55.3)
Vomiting of everything	Yes	171(35.6)
	No	310 (64.4)
Convulsion	Yes	47(9.8)
	No	434(90.2)
Lethargy /unconsciousness	Yes	27 (5.6)
	No	454(94.4)
Shock	Yes	13 (2.7)
	No	468 (97.3)
Severe acute malnutrition	Yes	150(31.2)
	No	331(68.8)

**Clinical characteristics at admission**: Most (300, 62.4%) of the patients were ill for three or more days while nearly three fourth (347, 72.1%) of the children had their last meal less than eight hours before arrival. Over half (256, 53.2%) of the children had inability to drink or eat (Table 3). Over two third of the children had assessment of either severe pneumonia (181, 37.6%) or severe acute malnutrition (158, 32.8%) (Table 4).

**Table 3:** Clinical characteristics of critically ill children at admission to JMC, Southwest Ethiopia, 2019 (N=481)

Clinical characteristics on arrival	Category	Frequency (N, %)
Durations of illness in hour	<24 hours	104(21.6)
	24–71.99 hours	77(16.0)
	72–167.99 hours	147(30.6)
	≥168 hours	153(31.8)
Duration of last meal in hour	<2 hours	229(47.6)
	2–3.99 hours	40(8.3)
	4–7.99 hours	78(16.2)
	≥8 hours	134(27.9)
Vomiting on day of admission	Yes	215(44.7)
	No	266(55.3)
Diarrhea on the day of admission	Yes	147(30.6)
	No	334(69.4)
Inability to drink /eat	Yes	256(53.2)
	No	225(46.8)
Respiratory distress	Yes	385 (80.0)
	No	96(20.0)
Mental status	Impaired mental status	49(10.2)
	Conscious/alert	432(89.8)
Fever at admission	Yes	356(74.0)
	No	125(26.0)
Oxygen saturation at admission	<90	213(44.3)
	>90	268(55.7)
Seizure/ Convulsion at admission	Yes	53(11.0)

**Table 4:** Working diagnosis of critically ill children at admission to JMC, Southwest Ethiopia, 2019 (more than one condition possible)

Assessment	Frequency	Percentages
**Malaria**	7	1.5
**Severe pneumonia**	181	37.6
**Wheezing disorder**	34	7.1
**Sepsis**	26	5.4
**Severe acute malnutrition**	158	32.8
**Dehydration**	78	16.2
**Shock**	7	1.5
**Meningitis**	40	8.3
**Congestive heart failure**	50	10.4
**Severe anemia**	26	5.4
**Acute abdomen**	24	5.0
**Musculoskeletal infection**	4	0.8
**Others**	82	17.0

**Glycemic status:** Over a quarter of the children (139, 28.9%) had dysglycemia at admission, out of which, 24(5%) were hypoglycemic and 115(23.9%) were hyperglycemic. Almost equal proportion (135, 28.3%) of the children were also having dysglycemia after four hours of admission, out of which 21(4.4%) were hypoglycemic and 115 (23.9%) were hyperglycemic.

**Glycemic status and outcome of patients:** Out of 481 patients enrolled into the study, 27(5.6%) had died, out of which 9(1.9%) and 7(1.5%) had hypoglycemia and hyperglycemia at admission respectively. The proportion of death in those with dysglycemia (16/139, 11.5%) at admission was higher than those with euglycemia (11/342, 3.2%).

**Factors associated with dysglycemia at admission**: As is shown in Table 5 below, presence of sever acute malnutrition and altered level of consciousness were associated with hypoglycemia admission whereas residence out of Jimma Town and presence of diarrhea on date of admission were associated with hyperglycemia at admission.

**Table 5:** Multinomial logistic regression analysis of factors associated with dysglycemia at admission among critically ill children admitted to JMC, Jimma, South West Ethiopia, 2019

Variables Category		Glycemic status at admission	P value	COR(CI)	P- value	AOR(CI)
**Severe acute**	Yes	Hypoglycemia	0.02	**2.63 (1.14–6.1)**	0.02	**3.09(1.18–7.77)**
**malnutrition**	No		1	1	1	1
	Yes	Hyperglycemia	0.41	0.82(0.51–1.31)	0.99	0.99(0.59–1.70)
	No		1	1	1	1
**Residence of**	Jimma Town	Hypoglycemia	1	1	1	1
**the care giver**	Outside of Jimma		0.53	1.32(0.55–3.17	0.87	1.08(0.44–2.66)
	Jimma Town	Hyperglycemia	1	1	1	1
	Out of Jimma		0.01	**1.79(1.12–2.85)**	0.01	**1.85(1.15–2.96)**
**Diarrhea at**	Yes	Hypoglycemia	0.66	1.21(0.52–2.86)	0.88	0.93(0.34–2.50)
**admission**	No		1	1	1	1
	Yes	Hyperglycemia	0.02	**0.56(0.34–0.93)**	0.03	**0.54(0.31–0.95)**
	No					
Mental status	Impaired	Hypoglycemia	0.004	**4.13(1.59–10.73)**	0.003	**4.63(1.68–12.71)**
	Alert		1	1	1	1
	Impaired	Hyperglycemia	0.87	1.06(0.52–2.19)	0.78	0.90(0.43–1.88)
	Alert		1	1	1	1

**Factors associated with mortality**: On bivariate logistic regression analysis, children who were dysglycemic at admission were eighteen times more likely to die as compared to children who were euglycemic at admission (p <0.00, COR=18.8) but upon multivariate analysis, there was no significant association seen (p=0.68, 95%CI 0.19–2.94, AOR=0.76). On the other hand, children who were dysglycemic four hours after admission were four times more likely to die as compared to children who were euglycemic four hours after admission both on bivariate (p=0.004, COR=4.02, 95%CI1.54–10.47) and multivariate logistic regression analysis (p=0.007, AOR 6.26, 95%CI 1.63–23.96) (Table 6).

**Table 6:** Factors associated with mortality of critically ill children, JMC, 2019

Variables Category		Outcome of the patient	p-value	COR(CI)	p-value for AOR	AOR(CI)
**Dysglycemia**	Hypoglycemia	Death	**0.00**	**18.8(6.67–53.94)**	0.68	0.76(0.19–2.94)
**at admission**	Hyperglycemia					
			0.15	2.03(0.77–5.39)	0.23	3.70(0.4530.66)
	Euglycemia		**1**	1	**1**	1
	Hypoglycemia	Unknown ©	0.61	1.49(0.32–6.90)	0.21	1.76(0.73–4.25)
	Hyperglycemia		0.29	1.44(0.74–2.82)	0.47	2.05(0.29–14.15)
	Euglycemia		**1**	1	**1**	1
**Dysglycemia**	Hypoglycemia	Death	**0.004**	**4.02(1.54–10.47)**	**0.007**	**6.26(1.63–23.96)**
**at 4 hours**	Hyperglycemia		**0.00**	**30.89(10.01–95.23)**	0.03	**10.9(1.28–94.53)**
	Euglycemia		**1**	1	**1**	1
	Hypoglycemia	©Unknown	0.98	1.01(0.49–2.07)	0.51	0.73(0.29–1.87)
	Hyperglycemia		0.82	0.78(0.98–6.26)	0.58	0.48(0.04–6.48)
	Euglycemia		**1**	**1**	**1**	1
**Seizure at**	Yes	Death	**0.02**	**3.04(1.22–7.62)**	0.18	2.23(0.70–7.14)
**admission**	No		**1**	1	**1**	1
	Yes	©Unknown	0.69	0.81(0.28–2.36)	0.63	0.76(0.26–2.28)
	No		**1**	1	**1**	**1**
**Mental**	Impaired	Death	**0.00**	5.31(2.22–12.71)	**0.008**	**4.35(1.17–12.87)**
**status at**	Alert		**1**	1	**1**	1
**admission**	Impaired	©Unknown	0.64	1.27(0.47–3.47)	0.71	1.21(0.44–3.33)
	Alert		**1**	1	**1**	1
**Shock at**	Yes	Death	**0.001**	**16.83(3.23–87.87)**	**0.05**	**7.35(1.03–55.13)**
**admission**	No		**1**	**1**	**1**	1
	Yes	©Unknown	0.36	2.93(0.30–28.73)	0.46	2.40(0.23–24.50)
	No		**1**	**1**	**1**	**1**
**Sepsis at**	Yes	Death	**0.03**	**3.76(1.18–12.02)**	**0.02**	**5.25(1.25–22.09)**
**admission**	No		**1**	1	**1**	**1**
	Yes	Unknown	0.23	2.01(0.65–6.21)	0.18	2.20(0.70–6.95)
	No		**1**	1	**1**	**1**
**Acute**	Yes	Death	**0.01**	**5.94(1.98–17.83)**	**0.002**	**8.04(2.50–29.81)**
**abdomen at**	No		**1**	1	**1**	1
**admission**	Yes	Unknown	0.13	2.43(0.77–7.66)	0.12	2.50(0.78–8.02)
	No		**1**	1	**1**	1

©Unknown include patients who left against medical advice and who disappeared from treatment before they finished their course of treatment

In the present study, the prevalence of dysglycemia at admission in critically ill children was 28.9%. This finding was comparable with findings reported from studies done in Mozambique, Laos, Cote d'Ivoire, Madagascar, Nigeria-Osun and Kenya (4,9,14,15). However, it is much higher than another study done previously in Jimma Medical Centre which has shown the prevalence of dysglycemia to be 6.0% (13). The possible reasons for this difference could be the difference in the method used including the study subjects (all patients visiting EOPD instead of critically ill patients were included) and the background characteristics of the participants.

In our current study, children who had severe acute malnutrition, children who came with altered state of consciousness and children who came out of Jimma Town were at higher risk to have dysglycemia as compared to those with euglycemia. Children who had severe acute malnutrition were three times more likely to be dysglycemic as compared to children who did not have malnutrion. This has been demonstrated in other studies as well, and it may be due to the impairment of blood-glucose homeostasis in children who have severe acute malnutrition necessitating serious attention to be paid to early detection, prevention and treatment of dysglycemia in children with severe acute malnutrition (16,17). An intact energy balance and maintenance of normal blood sugar concentration is dependent upon an adequate caloric and qualitative dietary intake; a functionally intact hepatic glucogenolytic and gluconeogenic enzy me system; an adequate supply of endogenous gluconeogenic substrates (lactate, amino acids and glycerol); an adequate energy supply provided by the beta-oxidation of fatty acids to synthesize glucose and ketone bodies and a normal endocrine system (insulin, glucagon, catecholamines and growth hormone) for integrating and modulating these processes. As these factors are disrupted in children with severe acute malnutrion, increased risk of dysglycemia could be related to these factors (18).

Children who came from outside Jimma Town were almost two times more likely to be dysglycemic as compared to children who came from Jimma Town. This may be due to delay in presentation to the hospital, and prolonged delay of referral before diagnosis was made, which lead such children to have critical illness which intern leads to stress hyperglycemia. Similarly, children who had altered state of consciousness were at higher risk to have dysglycemia (hypoglycemia specificall) as compared to those who did not have altered state of consciousness. This could be due the fact that children with altered state of consciousness may have inability to feed, and they could have severe illness. A research done in Mozambique also showed unconsciousness and malnutrition to be independent risk factors for hypoglycemia (19).

In our current study, children who were dysglycemic at admission and four hours after admission were found to be at high risk of death as compared to children who were euglycemic. Similar to our finding, a research done in PICU in Iran demonstrated that children who were dysglymic were found to be at higher risk to die as compared to children who were euglycemic (20). Another study also showed that dysglycemia is linked with a high risk of mortality for children in non-malaria tropical settings (4). A Similar other study done in Ghana showed that subjects with dysglycaemia were 3 times more likely to die and 4.8 times more likely to have complications than those with euglycemia (5).

To the best of our knowledge, the present study is the first of its kind to determine the magnitude of dysglycemia, associated factors and outcome of children with dysglycemia in the setting which can be taken as the strength of the study with dysglycemia among critically ill children in the study area. However, our result should be interpreted in the light of some limitations. We only included children who come to seek medical care which may not represent all cases. Caution, therefore, is needed in interpreting our results as it needs confirmation using large cohort in a multi-center study. Another limitation of our study is that we did not calculate sample size; rather, we consecutively included all critically ill children who fulfilled the inclusion criteria during the study period.

In conclusion, dysglycemia was found to be a common problem in critically ill children in this study. Hyperglycemia is relatively frequently seen in critically ill children than hypoglycemia. Dysglycemia at admission and four hours after admission was significantly associated with death. Blood glucose determination should be done for all critically ill children on arrival to the hospital and managed as early as possible. Empiric administration of dextrose to critically ill children should be minimized. Further multi center study overcoming the limitations of the current study is also recommended.

## References

[1] Guideline U (2016). Paediatric Emrgency Triage, Assessment and Treatment.

[2] Organization WH (2014). IMCI chart booklet.

[3] Anand P, Taneja V, Chugh K (2017). Glycemic Control in Critically Ill: A Review.

[4] Barennes H, Sayavong E, Pussard E (2016). High mortality risk in hypoglycemic and dysglycemic children admitted at a referral hospital in a non malaria tropical setting of a low income country. PloS one.

[5] Ameyaw E, Amponsah-Achiano K, Yamoah P, Chanoine J-P (2014). Abnormal blood glucose as a prognostic factor for adverse clinical outcome in children admitted to the paediatric emergency unit at Komfo Anokye Teaching Hospital, Kumasi, Ghana. International journal of pediatrics.

[6] Van Waardenburg DA, Jansen TC, Vos GD, Buurman WA (2006). Hyperglycemia in children with meningococcal sepsis and septic shock: the relation between plasma levels of insulin and inflammatory mediators. The Journal of Clinical Endocrinology & Metabolism.

[7] Kamińska H, Wieczorek P, Skała-Zamorowska E, Deja G, Jarosz-Chobot P (2016). Dysglycemia in critically ill children. Pediatric Endocrinology, Diabetes & Metabolism.

[8] Diana Grace R (2015). Clinical Profile and outcome of stress hyperglycemia in critically ill children.

[9] Sambany E, Pussard E, Rajaonarivo C, Raobijaona H, Barennes H (2013). Childhood dysglycemia: prevalence and outcome in a referral hospital. PLoS One.

[10] Elusiyan J, Adejuyigbe E, Adeodu O (2005). Hypoglycaemia in a Nigerian paediatric emergency ward. Journal of tropical pediatrics.

[11] Toro-Polo LM, Ortiz-Lozada RY, Chang-Grozo SL, Hernandez AV, Escalante-Kanashiro R, Solari-Zerpa L (2018). Glycemia upon admission and mortality in a pediatric intensive care unit. Revista Brasileira de Terapia Intensiva.

[12] Save SU, Ahmed M, Suri A (2016). Alteration of Homeostasis: Prevalence, Effect of Therapy and Outcome in Pediatric Intensive Care Unit at a Tertiary Care Centre. Journal of Contemporary Medical Research.

[13] Ruffo M, Workneh N (2012). Blood Glucose Levels in Pediatric Emergency Admission in Jimma University Specialized Hospital, Southwest Ethiopia. Ethiopian Journal of Pediatrics and Child Health.

[14] Wintergerst KA, Buckingham B, Gandrud L, Wong BJ, Kache S, Wilson DM (2006). Association of hypoglycemia, hyperglycemia, and glucose variability with morbidity and death in the pediatric intensive care unit. Pediatrics.

[15] Lou S, MacLaren G, Paul E, Best D, Delzoppo C, Butt W (2015). Prevalence of dysglycemia and association with outcomes in pediatric extracorporeal membrane oxygenation. Pediatric Critical Care Medicine.

[16] Wharton B (1970). Hypoglycaemia in children with kwashiorkor. The Lancet.

[17] Chisti MJ, Ahmed T, Ashraf H, Farugue A, Huq S, Hossain MI (2011). Hypoglycemia in children attending the critical care medicine in developing countries. Rigobelo EC, editoers Diabetes: Damages and Treatment.

[18] Nuoffer J, Mullis P (2005). Hypoglycaemia--diagnosis and therapy in emergencies. Therapeutische Umschau Revue Therapeutique.

[19] Madrid L, Acacio S, Nhampossa T, Lanaspa M, Sitoe A, Maculuve SA (2016). Hypoglycemia and risk factors for death in 13 years of pediatric admissions in Mozambique. The American journal of tropical medicine and hygiene.

[20] Khajavi L, Khademi G, Mehramiz M, Norouzy A, Safarian M (2018). Association of dysglycemia with mortality in children receiving parenteral nutrition in pediatric intensive care unit. Turkish Journal of Pediatrics.

